# The Protective Effect of Oxitard on Sperm Function and Antioxidant Status in Rats Exposed to Swimming Stress

**DOI:** 10.7759/cureus.40381

**Published:** 2023-06-13

**Authors:** Sangshetty Vijay Prashad, Kshatrapal Prajapati, Gurudatta Moharir, Nkemcho Ojeh, Susmita Sinha, Santosh Kumar, Mainul Haque, Ambadasu Bharatha

**Affiliations:** 1 Pharmacology, Shrimant Rajmata Vijayaraje Scindia Medical College and Hospital, Shivpuri , IND; 2 Community Medicine, Government Medical College, Shivpuri, IND; 3 Pharmacology and Therapeutics, Dr. Ulhas Patil Medical College and Hospital, Jalgaon, IND; 4 Preclinical and Health Science, Faculty of Medical Sciences, The University of West Indies, Cave Hill, BRB; 5 Physiology, Khulna City Medical College and Hospital, Khulna, BGD; 6 Periodontology and Implantology, Karnavati University, Gandhinagar, IND; 7 Karnavati Scientific Research Center (KSRC), School of Dentistry, Karnavati University, Gandhinagar, IND; 8 Pharmacology and Therapeutics, National Defence University of Malaysia, Kuala Lumpur, MYS; 9 Department of Preclinical and Health Sciences, The University of West Indies, Cave Hill, BRB

**Keywords:** albino rats, swimming stress, male fertility, activity, spermatozoa, mechanism, shielding, oxitard

## Abstract

Background: Infertility is a significant public health issue, but its impact on quality of life and treatment efficacy is limited. Modern medicine lacks safe and effective drugs for male infertility, while traditional medicine has explored herbal extracts like Oxitard, which contains multiple extracts and oils. This study aimed to investigate the effects of Oxitard on male rats exposed to swimming (SW) stress.

Methods: Albino rats weighing 220-250 g were divided into five groups: control, SW stress, and SW treated with Oxitard at low, medium, and high doses of 250, 500, and 750 mg/kg/day, respectively. The rats were subjected to SW stress for 15 days and then assessed for body weight, reproductive organ weight, testosterone, antioxidant status, sperm function, and histological changes in the testes, seminal vesicles, and vas deferens.

Results: The results showed that SW stress significantly reduced body weight, seminal vesicle weight, testosterone levels, superoxide dismutase (SOD), catalase (CAT), sperm count, sperm motility, sperm viability, and significantly increased malondialdehyde (MDA) levels. The testes of the SW-stress group rats also showed a significant decrease in spermatogenesis and the number of seminiferous tubules containing sperm. In contrast, treatment with Oxitard, especially at the highest dose, demonstrated potent free radical scavenging activity, recovering antioxidant status, and sperm function.

Conclusion: SW stress led to decreased sperm function, antioxidant status, and increased lipid peroxidation (LPO) in male rats. Oxitard treatment, particularly in high doses, showed a potential role as a free radical scavenger in treating oxidative stress (OS)-associated male infertility. Further studies are needed to investigate the individual components of Oxitard and conduct clinical trials in human subjects.

## Introduction

Infertility is a prevalent public health concern that not only affects individuals but also has a significant impact on their interpersonal relationships [1,2]. Despite male factors contributing to 20-25% of all causes of infertility, there is a widespread misconception that infertility is solely a female issue, particularly in patriarchal societies [3,4]. Recent research suggests that environmental and lifestyle factors, such as diet and its impact on antioxidant levels and oxidative stress (OS), may affect fertility [5,6]. The epidemiological evidence suggests that OS plays a crucial role in the etiology of male infertility [7].

The impact of diet and its connection to fertility is still not entirely comprehended, and additional research is required to fully grasp the role of antioxidants and OS in fertility [7]. Additionally, while numerous studies have been published examining the therapeutic effects of various restorative antioxidants in infertility, there is currently no consensus on their effectiveness [7].

The use of small experimental animals, such as the swimming (SW)-stress model, is a commonly employed method for studying the physiological effects of stress on an organism [8,9]. The SW-stress model involves exposing animals to swimming as a form of exercise, which has been shown to elicit a stress response in the organism [8,10]. Additionally, swimming has been shown to burn more calories than treadmill jogging for an equal amount of time [11].

Plant compounds, such as those found in Oxitard, a phytopharmaceutical formulation containing a combination of Mangifera indica Linn, Glycyrrhiza glabra Linn, Syzygium aromaticum Linn, Vitis vinifera Linn, and Withania somnifera, have the potential to affect a wide variety of cellular functions and have significant disease-fighting potential [12,13]. Oral administration of Oxitard has been shown to increase antioxidant status and reduce lipid peroxidation (LPO) [14,15]. This slowing down of oxidation reactions protects against damage caused by LPO or free radicals, thereby protecting vital organs and maintaining the individual's health. Oxitard has been reported to possess antioxidant, adaptogenic, gastric protective, cardioprotective, and immunomodulatory properties [15-17].

It should be noted that while Oxitard has been reported to have potential therapeutic effects, more research is necessary to fully understand its mechanism of action and potential uses as a treatment for various conditions. Additionally, it is important to conduct further studies to investigate the effectiveness of Oxitard in humans under controlled clinical settings.

Why is an animal study required?

Experimental animal models are crucial for understanding disease progression and developing new therapies [18]. Rodent species, such as rats, mice, and guinea pigs, have a high degree of homology with human genomes, making them valuable models for studying reproductive biology [19]. Additionally, the rapid aging of rats, with a human year equating to approximately 10 days of rat life, allows for efficient experimentation and rapid acquisition of results [20].

In this study, rats were utilized to assess the impact of the SW-stress paradigm on male reproductive processes. The study aimed to investigate the potential antioxidant effects of Oxitard on SW-stress-induced changes in male fertility to explore previously unexamined areas of infertility research.

## Materials and methods

Study design

This study employed an experimental animal-based design.

Ethical Approval

The research was approved by the Institutional Animal Ethics Committee (IAEC) of B J. Medical College and Sassoon General Hospitals, Pune, India, with reference number BJMC/IEC/Pharmac/D 1210124-26 dated December 23, 2010. The study followed the animal welfare guidelines set by the Committee for Control and Supervision of Experiments on Animals (CPCSEA).

Inclusion Criteria

We acquired adult male albino rats weighing 220-250 g and aged 9-12 weeks from an authorized breeding center. The rats were placed in wire-bottom cages for one week to acclimate. They had tap water ad libitum throughout this period and were fed a well-balanced diet. The rats were kept in a controlled environment with a 12-hour light-dark cycle at 25°C [21].

Exclusion Criteria

Rats displaying abnormal behavior, anomalies, and violent activities were excluded from the study.

Sample Size

The study included N=6 animals per group under Schedule Y of the Drug and Cosmetic Act [22].

Grouping

Rats were divided into five groups of six animals. These groups were control, SW-stress, SW-stress plus low-dose Oxitard, SW-stress plus medium-dose Oxitard, SW-stress plus high-dose Oxitard (Table 1).

**Table 1:** Grouping of animals in the study

Group	Treatment
Control	None
Stress	SW-stress
Low-dose Oxitard	SW-stress + Oxitard 250 mg/kg/day
Medium-dose Oxitard	SW-stress + Oxitard 500 mg/kg/day
High-dose Oxitard	SW-stress + Oxitard 750 mg/kg/day

Drug Information

The pure granule form of Oxitard (available in the market as Oxitard capsules) was obtained from the Himalaya Drug Co. R&D Center in Makali, Bengaluru, India. Phytochemical and toxicological studies for the drug have been previously established. The powder form of the granules was administered orally via gavage using carboxymethyl cellulose as the vehicle.

Study Variables

Independent variables: SW-stress; dependent variables: Body weight, reproductive organ weight, testosterone levels, antioxidants (malondialdehyde (MDA), superoxide dismutase (SOD), catalase (CAT)), sperm function (count, motility, and viability), testicular index (TI), copulation index, tubular differentiation index (TDI), and histological changes in testes, seminal vesicles, and vas deferens.

Stress Method

The SW-stress method, as further modified by Porsolt et al. and Nayanatara et al., was used in this experiment [8,23,24]. Rats were exposed to swimming in plastic tanks (length 100 cm, depth 60 cm, and width 40 cm) containing tap water (temperature 36±2°C) at the same time (9 a.m. to 10 a.m.) until exhaustion (inability to rise to the surface to breathe within seven seconds) for 15 days.

Investigated Parameters

Body weight was measured on the first and sixteenth day using an electronic balance (BA 210s, D=0.0001 (maximum 1 kg)). Following the end of the study, the blood samples were collected, and the serum was extracted by centrifugation at 3,000 rpm for 20 minutes for biochemical analysis. testosterone levels were measured using ELISA kits [25]. At the end of the experiments, the testes, seminal vesicles, vas deferens, and cauda epididymis were removed, cleaned of any extra tissue, and used for histopathological and seminal examination.

Biochemical Analysis

Testicular Homogenate Preparation for Biochemical Assay: Testicular tissue (1 g) was suspended in a 10% w/v phosphate buffer with a pH of 7.4. Then the tissue was homogenized using a manual homogenizer, and broken cell debris was removed by centrifugation at 3,000 rpm for 10 min at -4°C. The obtained supernatant was stored at -80°C and used for the following antioxidant estimations [26-28].

SOD: SOD activity was measured using the method [29,30]. Absorbance was measured at 560 nm and expressed in units per milliliter of protein.

MDA: The MDA level, an indicator of LPO, was determined calorimetrically using the method described by Saalu et al. [31]. The absorbance was assessed at 535 nm using a reference blank, and the testosterone concentration was reported in units of nmol/mg protein.

CAT: The activity of CAT was measured using the method [32]. Spectrophotometric measurements of absorbance fluctuations at 240 nm were used to determine the rate of H2O2 degradation, and the activity was measured in units per ml.

Epididymal Sperm Parameters

Semen Samples: The cauda epididymis was homogenized using a manual homogenizer in 1 ml phosphate buffer solution (pH 7.2). The sperm were examined using the aliquot [33]. Sperm motility, viability, and sperm count were evaluated microscopically using the methods [27,34-36]

Seminal Fluid: Spermatozoa are collected from the vas deferens. Sperm motility, viability, and sperm count (standard Neubauer chamber method) were examed microscopically [26,34,37,38].

TI: Each rat was weighed at the end of the experiment. The accessory tissue of the left testis was removed and weighed [39]. The formula: TI = (Weight of Left Testicle/Body Weight) X 100. 

Study on Libido

Male rats were housed with three female rats (1:3) for 24 hours. At the end of this time, a vaginal swab was taken. The swab was diluted in normal saline and examined under a microscope. The presence of spermatozoa indicated that rats were copulating. The copulation index (CI) was calculated using the following formula: CI = (The number of female rats mated/Total number of female rats housed) X 100 [40].

TDI

The proportion of seminiferous tubules with three or more layers of differentiated germ cells, starting from the spermatogonial layer, was calculated as described [41].

Histopathological Parameters

The testes, seminal vesicles, and vas deferens tissues were collected for histological examination. The tissue samples were preserved in buffered formalin and subsequently embedded in paraffin. Sections of 5µm thickness were cut and stained using the conventional hematoxylin and eosin (H&E) [42,43].

Statistical analysis

Groups tabulated the data, and the parameters were written as a series in the methods section. SPSS version 16.0 (SPSS Inc., Chicago, USA) was used for data analysis. Post hoc tests and one-way ANOVA were used for comparison between groups.

## Results

At the end of the study, on day 16, the group subjected to SW stress exhibited a significant decrease in whole-body weight compared to the control group (p<0.001). Treatment with high doses of Oxitard resulted in recovery, but not to the level of the control group. SW stress also resulted in a significant decrease in both testes and seminal vesicle weights compared to the control group. However, treatment with high doses of Oxitard resulted in recovery to control levels (Table 2).

**Table 2:** Effect of SW-stress on organ indices *Comparison between control and all other groups; ^#^Comparison to stress group; *^,#^P <0.05; **P <0.01, ***^,###^P <0.001 Data were presented as mean±SD the test drug dose: per kg/day SW: Swimming

Group 1 (Control)	Group 2 (SW stress)	Group 3 (Oxitard 250 mg)	Group 4 (Oxitard 500 mg)	Group 5 (Oxitard 750 mg)
+5.33±5.61	-16.5±3.73***	-14.67±1.03 ***	-13±2.53***	-12.67±1.97***
1.07±0.06	1.001±0.01*	1.02±0.02	1.03±0.05	1.05±0.05
0.73±0.05	0.597±0.11***	0.638±0.02**	0.682±0.017^#^	0.703±0.01^###^
0.11±0.01	0.096±0.004***	0.101±0.003	0.103±0.003	0.106±0.002^#^

The findings from this study indicate that SW-stress decreased testosterone levels compared to the control group, which was statistically significant (p<0.05). However, this stress paradigm did not significantly affect the testicular or copulation index (p>0.05). Treatment with the high dose of Oxitard partially restored testosterone levels, although they did not fully recover to the levels observed in the control group p<0.05 (Table 3).

**Table 3:** Effect of SW-stress and Oxitard on hormonal indexes among experimental rats *P <0.001 compared to control Data were presented as mean±SD the test drug dose: per kg/day SW: Swimming; TI: Testicular index

Parameters	Group 1 (Control)	Group 2 (SW stress)	Group 3 (Oxitard 250mg)	Group 4 (Oxitard 500mg)	Group 5 (Oxitard 750mg)
Testosterone (ng/ml)	2.55±0.48	1.47±0.4*	1.5±0.16*	1.74±0.43*	1.96±0.15*
TI (%)	0.401±0.02	0.407±0.00	0.414±0.024	0.41±0.018	0.42±0.01
Copulatory index (%)	91.7±20.41	83.33±25.82	75±27.39	83.33±25.82	83.3±25.82

SW-stress significantly decreased CAT, SOD, and sperm function and significantly increased MDA levels compared to the control group (Table 4). This increase in MDA levels suggests that the rats were experiencing OS. Treatment with low and medium doses of Oxitard significantly improved the oxidative status. In contrast, the high dose of Oxitard significantly reduced the oxidative status to levels comparable to the control group (Table 4).

**Table 4:** Effect of SW-stress and Oxitard on antioxidants biomarkers *Comparison between control and all other groups; ^#^Comparison to stress group; ^£^Comparison between the low and medium dose of Oxitard; ^¥^Comparison between the low and high dose of Oxitard-treated group; ^†^Comparison between the medium and high dose of antioxidant Oxitard; ***^, ###, £££, ¥¥¥, †††^P <0.001 Data were presented as mean±SD the test drug dose: per kg/day SW: Swimming; SOD: Superoxide dismutase; MDA: Malondialdehyde; CAT: Catalase

Parameters	Group 1 (Control)	Group 2 (SW stress)	Group 3 (Oxitard 250 mg)	Group 4 (Oxitard 500 mg)	Group 5 (Oxitard 750 mg)
SOD (units/mg protein)	5.07±0.2	3.29±0.3***	3.33±0.26***	4.15±0.1***^###£££^	4.85±0.2^###¥¥¥††^
MDA (nmol/g tissue)	12.16±0.4	17.06±0.2***	15.63±0.47***^###^	13.93±0.17***^###£££^	12.8±0.6^###¥¥¥††^
CAT (nmol/g/tissue)	7.46±0.15	4.23±0.3***	4.40±0.39***	6.47±0.18***^###£££^	7±0.12^###¥¥¥^

In the current study, extreme SW stress significantly reduced sperm count, motility, and viability compared to the unstressed control group (P<0.001) (Table 5). These findings indicate that SW stress induces stress and alters male fertility. Comparison between the SW-stress group and the medium and high-dose groups pre-treated with Oxitard showed significant improvements in sperm count, motility, and viability. However, there remained a substantial difference between the control and high-dose groups.

**Table 5:** Effect of SW-stress and Oxitard on sperm parameters *Comparison between control and all other groups; ^#^Comparison to stress group; ^£^Comparison between the low and medium dose of Oxitard; ^¥^Comparison between the low and high dose of Oxitard-treated group; ^†^Comparison between the medium and high dose of antioxidant Oxitard; #P <0.05, ^££,¥¥,††^P <0.01; ***^,###,¥¥¥^P <0.001 Data were presented as mean±SD the test drug dose: per kg/day SW: Swimming

Parameters	Group 1 (Control)	Group 2 (SW stress)	Group 3 (Oxitard 250 mg)	Group 4 (Oxitard 500 mg)	Group 5 (Oxitard 750 mg)
Sperm count (Cu/mm/ epididymis)	778.3±21.4	589.5±31.8***	606.7±12.5***	660.8±20.1***^###££^	710±14.1***^###¥¥¥††^
Sperm motility (%)	80.3±3.14	66.3±3.83***	67.8±2.04***	70±1.1***	73.3±1.37***^###¥¥^
Sperm viability (%)	80±3.4	68±5.3***	67.7±1.6***	69±1.1***	73.3±1.2***^#¥¥^

Histopathological evaluation

**Figure 1:**
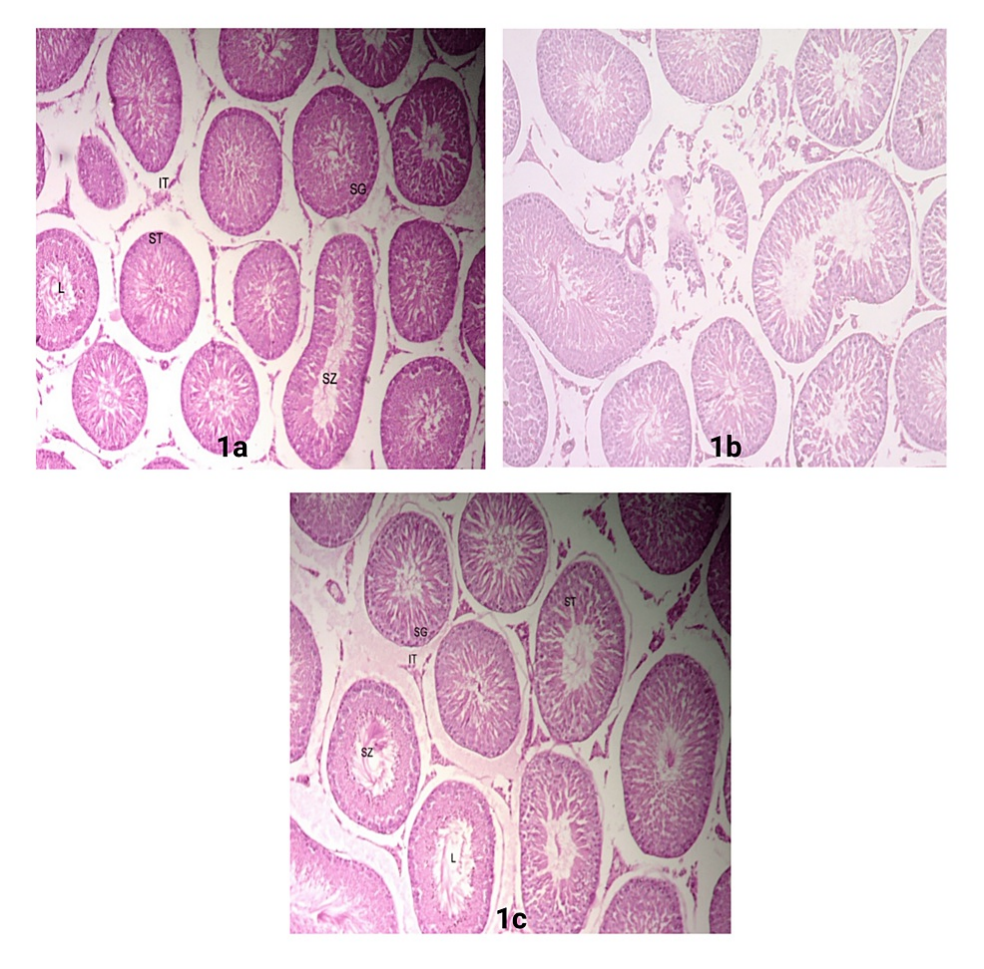
Histological features of rat testicular tissue in control, SW-stress, and Oxitard-treated SW-stress groups a: Testes of control rat; b: Effect of SW-stress on testicular tissue showing abnormal seminiferous tubule structure, lumen showing the arrest of sperm maturation, and a decrease in different stages of sperm; c: High dose of Oxitard treatment with normalization of the structure (magnification 40x) SW: Swimming; L: Lumen; ST: Seminiferous tubule; SG: Spermatogonia; SZ: Spermatozoa; IT: Interstitial tissue

Figure 3(a) depicts the typical architecture of the testicular tissue, characterized by the presence of seminiferous tubules, which are round and oval and surrounded by a basement membrane of myoid cells and a stratified epithelium. Sertoli cells comprise each tubule, and spermatozoa are arranged in rows between and around the cells. The seminiferous tubules displayed normal spermatogenesis with different stages of germ cells present. Spermatogonia gradually migrate to the middle of the tubules and transform into flagellated-free sperm. The lumen showed mature spermatids in its center. The seminiferous tubules interacted through the interstitial tissues (Figure 1a). In contrast, the testes of the SW-stress group rats had seminiferous tubules with reduced spermatogenesis, a decrease in the number of sperm-containing seminiferous tubules, and a lack of spermatozoa. An area of tissue destruction expanded the interstitial space. Some of the seminiferous tubules showed abnormal shapes. Some seminiferous tubules appeared after spermatogenic cells stopped maturing and were disorganized (Figure 1b). Treatment with the high dose of Oxitard on the SW-stress group restored the testicular architecture to normal (Figure 1c).

**Figure 2:**
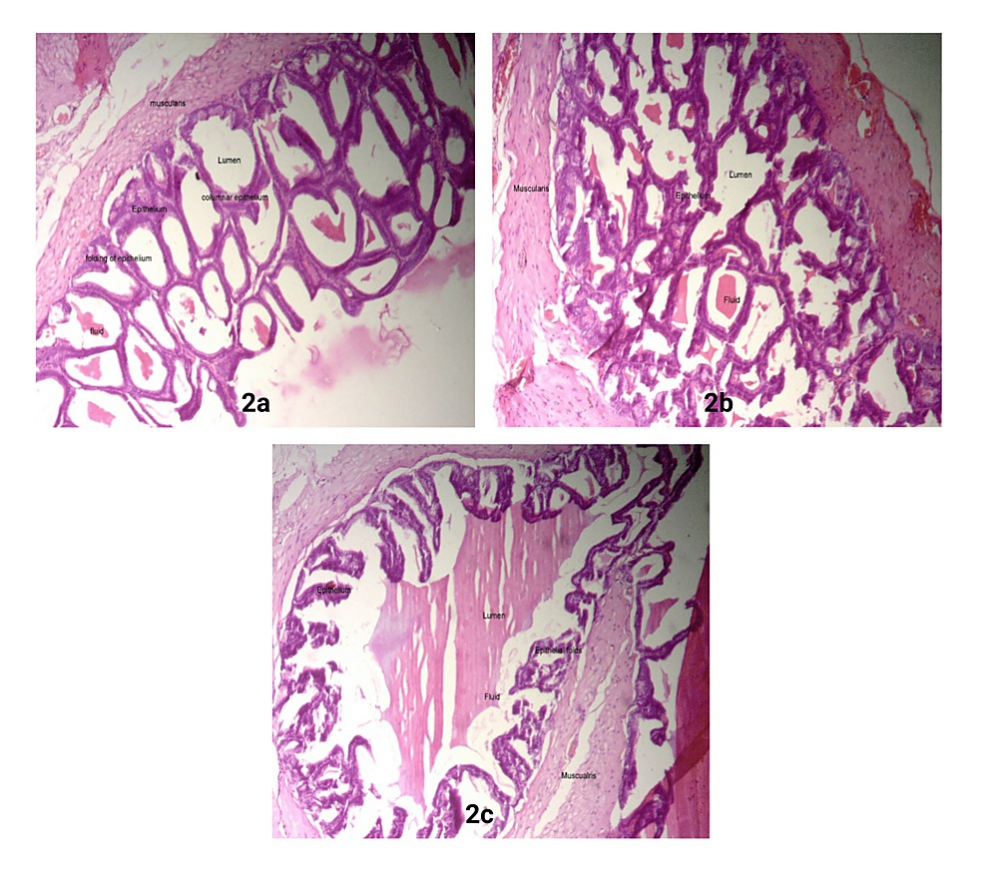
Histological features of rat seminal vesicles in Control, SW-stress, and Oxitard-treated SW-stress groups a: Normal seminal vesicle; b: Stress-induced changes in the seminal vesicle; c:  Oxitard-treated with high dose (40x) SW: Swimming

In the seminal vesicle of the control group, the columnar epithelium was observed between delicate fibers, stroma was present, and mucosal folds were compressed, forming pseudostratified columnar epithelium. The basement membrane was highly folded, resulting in an apical lumen filled with eosin fluid and aberrant crypts (Figure 2a). SW-stress caused epithelial hyperplasia that altered the histological aspects of the mucosa and reduced the number of glands. Additionally, inflammatory leukocyte cells infiltrated the muscular cells, resulting in degenerative changes and focal loss (Figure 2b). Pre-treatment with a high dose of Oxitard in the SW-stress group showed recovery of the basement membrane, lining epithelium, and folds, and the epithelium displayed typical seminal vesicle architecture (Figure 2c).

**Figure 3:**
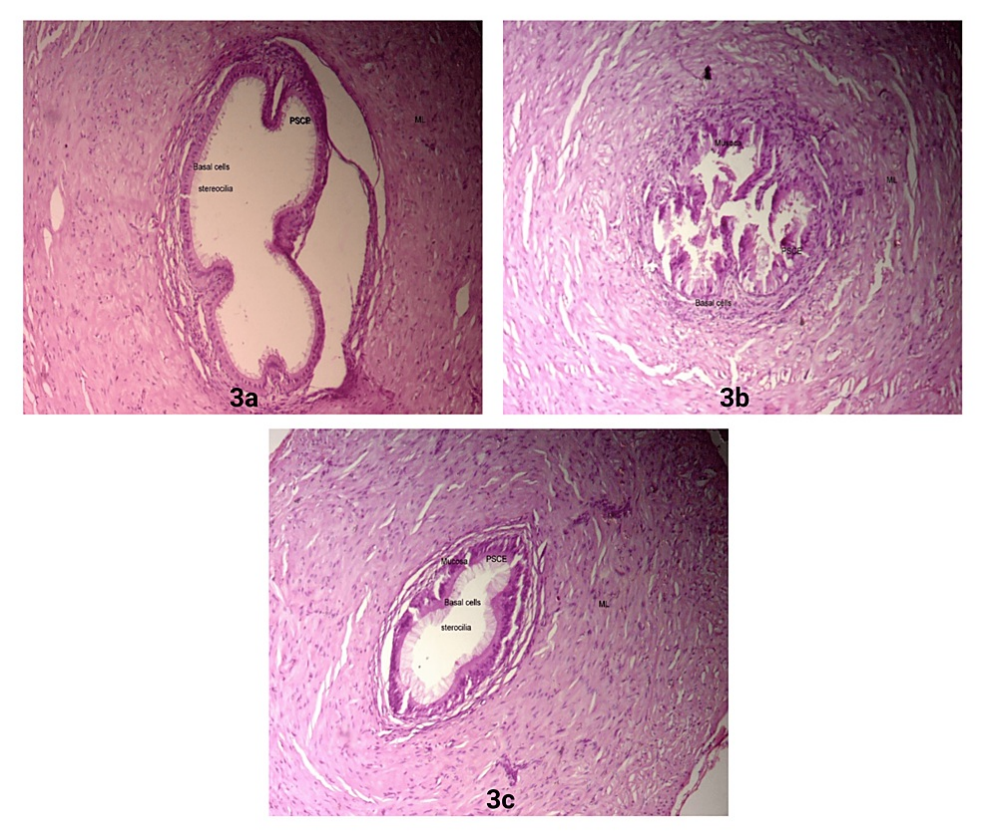
Histological features of rat vas deferens in Control, SW-stress, and Oxitard-treated SW-stress groups a: Normal vas deferens; b: Stress-induced changes in vas deferens; c: Oxitard-treated vas deferens (40x) SW: Swimming

In the vas deferens of the control group of rats, intricate mucosal folds and a compressed, slit-like lumen bordered by pseudostratified ciliated epithelium were observed. The basal layer contained columnar ciliated cells, and the muscular layer was thick. The lumen was empty, and the muscular coat, basement membrane, and lining epithelium remained normal, as seen in Figure 3a [39,40]. When the vas deferens were exposed to the SW-stress model, it revealed desquamated lining epithelium with atrophic changes, moderately exploited epithelium, and a degraded basement membrane, as seen in Figure 3b [14,44]. These changes were reversed in the Oxitard-treated group, as seen in Figure 3c [14,44].

## Discussion

**Figure 4:**
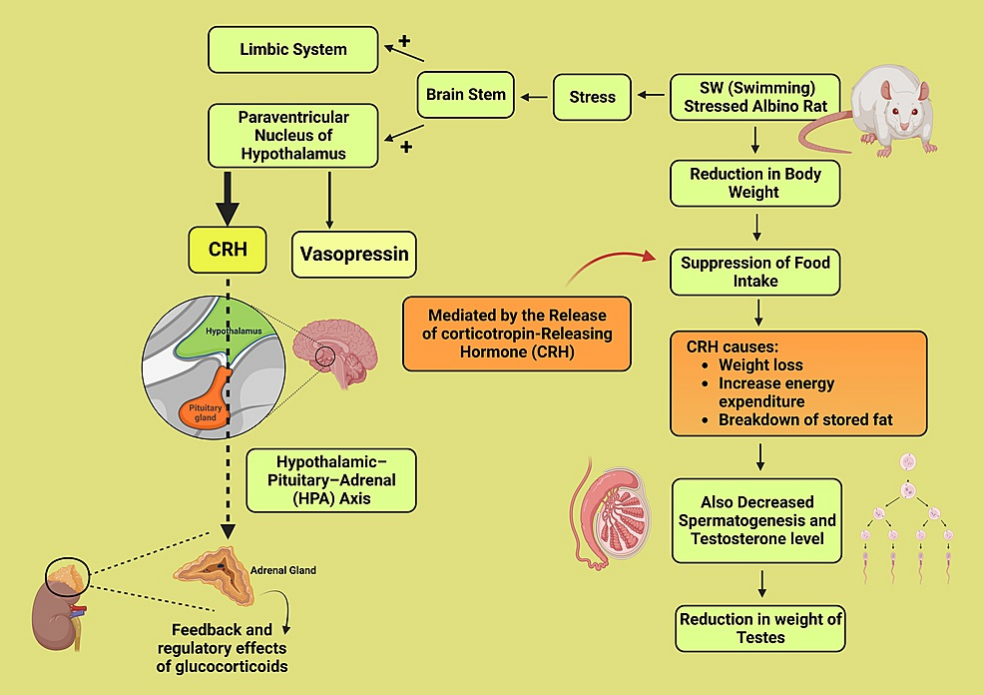
Schematic diagram showing the mechanism of weight reduction of the testes in the stressed group This figure has been drawn utilizing the premium version of BioRender with the License number IN25E8ZXYL. Image Credit: Susmita Sinha

The effect of SW stress on organ indices was evaluated in this study. Results showed that SW stress significantly reduced total body weight compared to the unstressed group (control group). The mechanism behind this reduction in body weight is thought to be related to the suppression of food intake, which is mediated by the release of corticotropin-releasing hormone (CRH) in response to stress. CRH is known to suppress appetite while increasing energy expenditure, leading to the breakdown of stored fat and cholesterol in the body, ultimately leading to weight loss. Additionally, the testes' weight was reduced due to the loss of spermatogenesis and decreased testosterone levels in the stressed group (Figure 4). However, pre-treatment with Oxitard significantly increased the weight of the testes, suggesting its anti-stress properties. These findings are consistent with multiple previous studies [13-16].

Effect on hormonal function

The stress stimulus likely acts on the gonadal axis, inhibiting gonadal function by producing glucocorticoids and catecholamines and activating CRH neurons. CRH has been shown to have an adverse regulatory effect on Leydig cells, decreasing testosterone biosynthesis [45-48]. Multiple studies reported that stress could lead to a decrease in sexual desire and transient impotence in men, potentially due to the release of a brain chemical that constricts the smooth muscle of the penis and its arteries [49-52]. In the present study, decreased testosterone levels (P<0.001) and sexual desire compared to the control group may indicate a similar effect. While the copulation index was reduced in the current study, it was not statistically significant (P>0.05). The antioxidant-mediated activity of Oxitard may explain its protective effects during stress-related hormonal changes [13].

Sperm function

Sperm cell membranes contain high concentrations of phospholipids, sterols, saturated and polyunsaturated fatty acids, making them more susceptible to damage from excessive reactive oxygen species (ROS) releases and resulting in disruption of the antioxidant status in sperm [53-57]. It leads to the onset of male infertility [3,57,58]. Elevated levels of MDA can lead to decreased sperm motility [59]. Free radicals (LPO) have been linked to male fertility by reducing sperm motility. The arrest of sperm motility is probably due to the ROS-induced cascade affecting sperm axoneme function [48,49].

**Figure 5:**
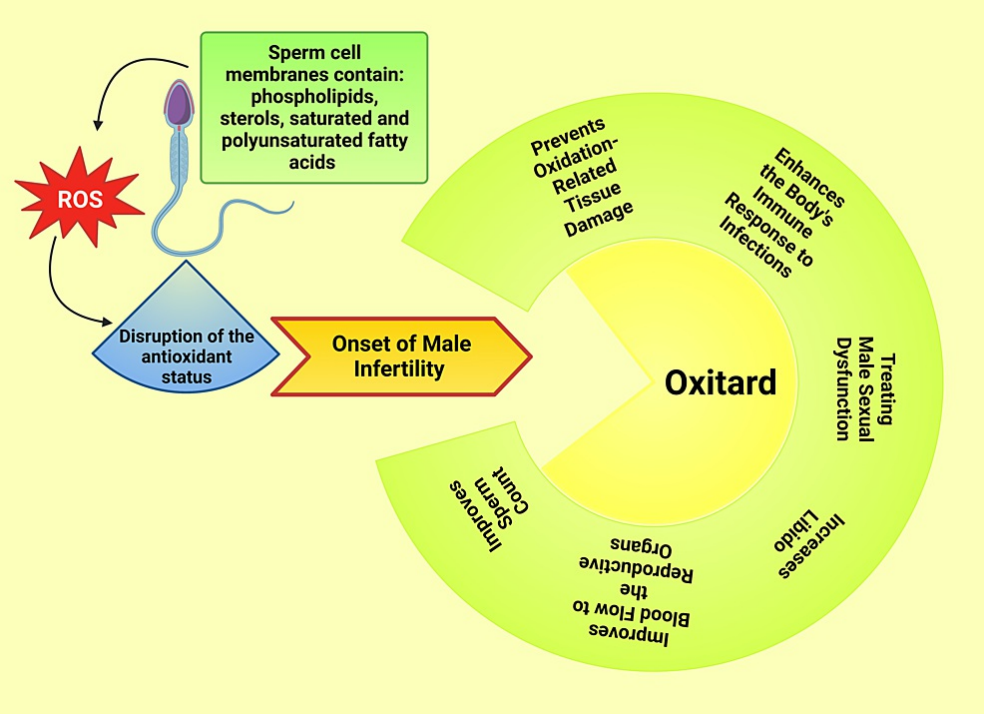
Illustration showing beneficial effects of oxitard on sperm function This figure has been drawn utilizing the premium version of BioRender with the License number XH25E9YT59. Image Credit: Susmita Sinha.

The decreased sperm count has been attributed to decreased reproductive hormones secreted by the hypothalamic-pituitary-testicular axis and associated with testosterone, gonadotropin-releasing, follicular stimulating, and luteinizing hormones [50]. In addition, changes in sperm viability due to changes in the microenvironment in the epididymis and increased LPO are unfavorable to the normal state of sperm [36]. A low number of sperm causes most male-factor reproductive problems, but motility plays a crucial role in achieving pregnancy [60,61]. Oxitard helps prevent oxidation-related tissue damage and enhances the body's immune response to infections, treating male sexual dysfunction, increasing libido, and improving sperm count. It improves blood flow to the reproductive organs by causing vasodilation (Figure 5), which can help maintain a firm erection long enough for intercourse [13,14].

Additionally, the effects of SW stress on the antioxidant status of testicular tissue were examined. Results showed a significant increase in MDA levels (P<0.001) and decreased levels of SOD and CAT (P<0.001) in testicular tissue of rats under stressed conditions. These findings are consistent with previous studies by Mrakic-Sposta et al., Pizzino et al., He et al., Oyeyipo et al., Lubkowska et al., and Lodhi et al. [62-67]. Oxitard pre-treatment improved OS parameters related to SW-stress in terms of increased SOD, CAT, and decreased MDA levels, indicating that the capacity of the antioxidant Oxitard increased due to its ability to scavenge and scavenge free radicals as well as its nourishing effects. These findings suggest that Oxitard may have protective benefits during stress-related hormonal changes and OS, which can lead to male infertility. Additional studies are required to comprehensively comprehend the mechanisms that underlie these effects.

Histopathology of male reproductive organs

The present study examined the effects of restraint stress on histopathological parameters of the testes, seminal vesicles, and vas deferens in a rat model. According to previous research, restraint stress can cause disorganization of the germinal epithelium, an increase in interstitial spaces, a reduction in the number of spermatogonial spermatids, and a decrease in the diameter of the seminiferous tubules, as well as poorly defined Leydig cells and smaller cell nuclei compared to controls [68, 69].

Antioxidants scavenge free radicals and bind metal at various stages of the oxidation process [70,71]. Ions are used to scavenge peroxyl radicals and eliminate oxidatively damaged biochemicals [72]. It is, therefore, a polyherbal Ayurvedic formulation. Oxitard's active granules showed an oxygen radical absorbance capacity (ORAC) value of 2084.9 mol TE/gram, indicating that each serving of Oxitard capsules contains ORAC units comparable to Vitamin C. Withania somnifera, Glycyrrhiza glabra, Syzygium aromaticum, and Vitis vinifera are free, potent antioxidants and inhibitors radicals that damage various organs and systems [73-81]. Although Emblica officinalis is known for having the highest Vitamin C content and is revered for its rejuvenating powers, most current scientific interest focuses on its unique tannins and flavonoids, which contain powerful antioxidant properties [82,83]. However, more studies indicate that Emblica officinalis can stimulate our natural antioxidant enzyme system, including CAT, SOD, and glutathione peroxidase [84,85]. Antioxidant enzymes such as SOD, glutathione peroxidase, and CAT and substances like glutathione eliminate free radicals and protect cells and tissues from oxidative attacks [86,87].

In this study, the test animals were pre-treated with Oxitard has been shown to stimulate the natural antioxidant enzyme system, including CAT, SOD, and glutathione peroxidase [88]. The study's results showed that restraint stress caused a significant increase in the levels of MDA and a decrease in the levels of SOD and CAT in the testicular tissue of the rats, indicatingOS [88-90]. However, pre-treatment with Oxitard, particularly at medium and high doses, significantly improved the antioxidant status and sperm function compared to the stress group [91,92]. Comparison of the high-dose Oxitard group with the control group showed a restoration of antioxidant status (SOD and MDA) and sperm function. These findings suggest specifically that Oxitard, a polyherbal Ayurvedic formulation, significantly improved the antioxidant status and sperm function in rats exposed to swimming stress.

The administration of Oxitard at medium and high doses resulted in a significant improvement in antioxidant status (as indicated by a decrease in MDA levels and an increase in superoxide dismutase [SOD] activity) and sperm function (as indicated by an increase in sperm count, motility, and viability) compared to the stress group (p<0.001). Comparison of the high-dose Oxitard group with the control group revealed a restoration of antioxidant status (SOD activity, p<0.01) and sperm function (p<0.01).

Limitations of the study

The study has limitations, including utilizing a polyherbal formulation, Oxitard, comprising multiple active molecules. The specific pharmacological properties observed cannot be attributed to a single compound or molecule, and further analysis is needed to identify the specific active compounds. Additionally, the current study is limited by a small sample size and a short period, making it an acute study. Thus, further clinical trials are necessary to establish the therapeutic potential of Oxitard in humans.

## Conclusions

Decreased sperm function, reduced antioxidant status, and increased LPO indicate that swimming stress harms fertility. Oxitard administered at a high dose restored the antioxidant state and sperm function, indicating an effective free-radical scavenging action. As a result, it can be used to treat the OS associated with male infertility. The present study demonstrated that swimming stress leads to a significant decrease in sperm function, a reduction in antioxidant status, and an increase in LPO. These findings indicate that SW stress harms fertility. However, when Oxitard was administered in a high dose, there was a significant restoration of the antioxidant state and sperm function. This suggests that Oxitard has an effective free-radical scavenging action and may help treat OS associated with male infertility.
